# Somatic and germline analysis of a familial Rothmund–Thomson syndrome in two siblings with osteosarcoma

**DOI:** 10.1038/s41525-020-00160-x

**Published:** 2020-12-04

**Authors:** Miriam Gutiérrez-Jimeno, Elena Panizo-Morgado, Ibon Tamayo, Mikel San Julián, Ana Catalán-Lambán, Marta M. Alonso, Ana Patiño-García

**Affiliations:** 1 Department of Pediatrics, University Clinic of Navarra, Pamplona, Spain; 2 Computational Biology Program, CIMA, Center for Applied Medical Research, Pamplona, Spain; 3 Department of Traumatology, University Clinic of Navarra, Pamplona, Spain; 4 Solid Tumor Program, CIMA, Center for Applied Medical Research and IdiSNA, Pamplona, Spain

**Keywords:** Bone cancer, Molecular medicine, Cancer genomics

## Abstract

Rothmund–Thomson syndrome (RTS) is characterized by a rash that begins in the first few months of life and eventually develops into poikiloderma. Associated symptoms are alterations in the teeth, sparse hair, thin eyebrows, lack of eyelashes, low stature, bone abnormalities, hematological illnesses, gastrointestinal disease, malnutrition, cataracts, and predisposition to cancer, principally to bone tumors and skin cancer. Diagnostic certitude is provided by a genetic study involving detection of pathogenic variants of the *RECQL4* gene. We hereby present a familiar case of RTS in two siblings from a Portuguese family, both diagnosed with osteosarcoma. Genomic analysis (203 genes) of both tumors as well as germline analysis of the *RECQL4* gene, thus confirming the syndrome in the family, have been performed. The relevance of clinical recognition of the hallmarks of the disease and thus early diagnosis with early intervention is highlighted.

## Introduction

Rothmund–Thomson syndrome (RTS) is an autosomal-recessive genodermatosis with clinical symptoms that develop in early infancy. Symptoms include cutaneous alterations (typically, facial erythema and poikiloderma), hair loss, cataracts, growth delay, skeletal anomalies, premature aging, and an inherited predisposition to cancer (mainly pediatric osteosarcoma)^1–5^. There are few cases described in the literature; most reported cases are from isolated individuals.

Patients who are at the highest risk of developing tumors are those with homozygous or compound heterozygous mutations at the *RECQL4* gene, which belongs to the RecQ helicase family^5^.

We describe the cases of two siblings with confirmed germline compound heterozygous *RECQL4* mutations and with diagnoses of osteosarcoma. The segregation of the mutations in the parents has been analyzed and genomic somatic analysis of both tumors performed.

## Results

### Description of the cases

#### Case #1

Case #1 is the first child (female) of non-consanguineous Portuguese parents. Delivery was at term after a normal pregnancy. Birth weight was 2880 g, and height 25 cm (<p3). Neurodevelopment was normal. The patient was initially studied because of her low height. Bone age corresponded to her chronological age, and insulin-like growth factor-1 (IGF-1) and IGF binding protein-3 (IGFBP-3) levels were normal. The patient was subsequently attended to for facial poikiloderma with telangiectasia. Limbs were treated with laser therapy. Bone X-rays revealed diffuse demineralization. Blood tests found vitamin D deficiency. Comparative genomic hybridization yielded normal results.

At 6 years of age, the patient was attended to at a referral center because, for the previous month, she had had a pain in her right knee that interfered with her sleep. On the basis of computed tomography (CT), X-ray, and nuclear magnetic resonance (MR) imaging and a biopsy, the diagnosis made was of osteosarcoma of the distal right femur, involving both the metaphysis and epiphysis, without distant metastatic disease. Neo-adjuvant chemotherapy with the EURAMOS1 regimen was initiated, and amputation was advised. This regimen consists of two cycles of intravenous methotrexate (12 g/m^2^), Adriamycin (75 mg/m^2^), and cisplatin (120 mg/m^2^) before surgery (MAP therapy). Since the parents wanted to avoid amputation, they switched treatment center. In our center, treatment proceeded with preoperative intra-arterial cisplatin (150 mg/m^2^) and intravenous Adriamycin (100 mg/m^2^) plus vincristine (one dose of 1 mg). After neo-adjuvant treatment, limb-sparing surgery, consisting of tumor resection with distal femur reconstruction with an osteoarticular allograft, was performed. The pathology of the resected tumor was consistent with osteoblastic osteosarcoma of the distal femur with 95% tumor necrosis. Adjuvant chemotherapy was not administered because of the high degree of hematologic toxicity (grade 4 febrile neutropenia) of the neo-adjuvant treatment and because, as a result of receiving additional cycles of neo-adjuvant chemotherapy, the patient had already been subjected to higher final concentrations of anti-neoplastic drugs than those received by other patients under the same regimen.

The patient has been in clinical remission since the end of treatment (3 years and 4 months). She uses a 6.5 cm lift to compensate for bone length dysmetria due to the pathologic fracture of her sarcoma (previous to tumor resection). Further surgery will be required in the future.

#### Case #2

Case #2 is the younger brother of case #1. Medical examinations and tests revealed nothing of relevance except for growth delay (less than the third percentile, −3SD) despite normal IGF-1 and IGBP-3 levels. The patient was macrocephalic, but by brain MR there were no major alterations. He was diagnosed with celiac disease. Anti-peroxidase and anti-thyroglobulin antibody tests were positive. Thyroid function and thyroid echography were normal. He tested positive for anti-transglutaminase immunoglobulin A (IgA) and IgG.

At 5 years of age, he was attended to in our institution, where MR and CT imaging revealed cysts in the right femur and tibia. There was no indication that these cysts were aggressive. Three months later, however, he complained of pain in the medial face of the proximal right tibia. A biopsy was obtained, and a diagnosis of osteoblastic osteosarcoma of the proximal tibia was established. Additional tests were performed with no evidence of distant metastatic disease. Neo-adjuvant chemotherapy was initiated, with three cycles of intra-arterial cisplatin and intravenous Adriamycin. The tumor was resected. Pathologically, the resected piece was a 100% necrotic osteoblastic osteosarcoma. Adjuvant chemotherapy was initiated according to the MAP protocol, but, due to the toxicity of high-dose methotrexate, the patient suffered acute liver failure (hypertransaminasemia, hyperbilirubinemia, and coagulopathy), which required treatment with cholestyramine and activated charcoal, and thus chemotherapy was changed to cisplatin with Adriamycin.

The patient was in remission from February to August 2020, when lung metastases (two nodules in the left lung) were detected during scheduled follow-up. Further tests and lung metastasectomy are pending.

Table 1 gives the clinical characteristics of both patients with respect to the clinical signs indicative of a diagnosis of RTS.

**Table 1:** Clinical characteristics of our patients in relation to the clinical signs of RTS.

Clinical sign	Case #1 (sister)	Case #2 (brother)
Erythema on the cheeks and face	Present	Present
Poikiloderma	Present	Present
Sparse scalp hair, eyelashes, and/or eyebrows	Present	Present
Small size, usually symmetric for height and weight	Present	Present
Gastrointestinal disturbance as a young child: chronic vomiting and diarrhea	Present	Present
Dental abnormalities: hypoplastic teeth, enamel defects, delayed tooth eruption	Absent	Absent
Nail abnormalities: dysplastic or poorly formed nails	Absent	Absent
Hyperkeratosis (soles of the feet)	Absent	Absent
Cataracts	Absent	Absent
Skeletal abnormalities: radial ray defects, ulnar defects, absent or hypoplastic patella, osteopenia, abnormal trabeculation	Present	Present
Cancers including skin cancers: basal cell carcinoma, squamous cell carcinoma, osteosarcoma	Present	Present

### Germline genetic analysis

Because both siblings had osteosarcoma, we gave genetic counseling and sequenced germline *RECQL4* genes to test for RTS.

**Fig. 1:**
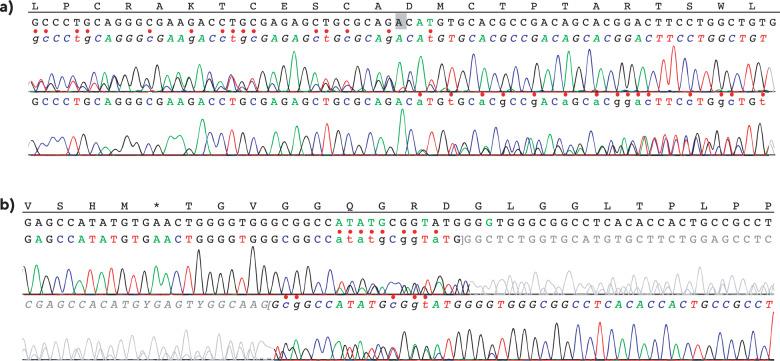
*RECQL4* mutation sequencing. Sanger sequencing of the germline *RECQL4* mutations present in both cases, showing the presence of mutations **a** c.2492_2493delAT, p.(His831Argfs*52) and **b** c.1878+32_1879-27del24 (p.(?)).

Both siblings were found to be compound heterozygotes for mutations c.1878+32_1879-27del24 (p.(?)) in intron 11 and c.2492_2493delAT (p.(His831Argfs*52)) in exon 15 of the *RECQL4* gene (Fig. 1). This finding establishes the diagnosis of RTS, given the autosomal and recessive nature of the disease.

Variant c.1878+32_1879-27del24, p(?): this variant in intron 11 of *RECQL4* potentially affects both the 5′-donor splice site and 3′-acceptor splice site of intron 11, which might result in aberrant mRNA splicing and consequently a non-functional protein. This intronic deletion, rs750811636, has been previously described in association with recessive RTS5^6,7^ and its frequency in the Exome Aggregation Consortium (ExAC) database is 3/236,462 (1/78,820, with no homozygotes).

Variant c.2492_2493delAT, p.(His831Argfs*52): this is a dinucleotide deletion that causes a frameshift from codon 831 and a premature stop 52 amino acids after the deletion. The mutation, rs752729755, is referenced in ClinVar as a pathogenic variant (RCV000006436) and, like the intron 11 mutation, has been previously described in RTS^8^. The frequency of this variant in the ExAC database is 16/208,088 (1/13,005, with no homozygotes).

Segregation studies demonstrated that c.2492_2493delAT (p.(His831Argfs*52) was inherited from the father and c.1878+32_1879-27del24 (p.(?)) from the mother.

**Fig. 2:**
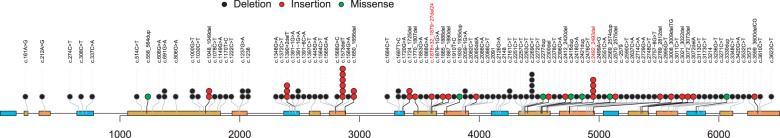
*RECQL4* mutation map. Mutation map of the RECQL4 gene, showing mutations in all exons. Deletions, insertions, and missense mutations are indicated with black, red, and green dots, respectively. All mutations are germline. Mutations found in our cases are in red lettering.

Figure 2 displays a mutation map of the *RECQL4* gene with the mutations that have been described so far, with those present in our patients highlighted in red.

### Somatic genomic analysis

The tumor of case #1 did not show any significant alteration in the genes of the Next-Generation Sequencing (NGS) panel. The tumor of case #2 was found to harbor a stop mutation in exon 17 of the *RB1* gene (NM_000321.2): c.1654C>T/p.(Arg552Ter). This mutation was sequenced at a depth of 1998× and the variant allele frequency in the tumor was 29%. The alteration has previously been reported to occur in retinoblastoma tumors as well as in patients with cancer syndromes^9,10^. The *RB1* gene is a tumor suppressor, located in 13q14, that codes for a protein that is a negative regulator of the cell cycle. *RB1* mutations are detected in between 10 and 60% of osteosarcoma tumors analyzed by NGS^11–13^.

## Discussion

The characteristics of type II RTS are poikiloderma and skeletal alterations and, genetically, homozygous or compound heterozygous mutations in the *RECQL4* gene. In contrast, type I RTS is characterized by juvenile cataracts and poikiloderma, and *RECQL4* mutations are not usually present.

To the best of our knowledge, the current report is only the second report of a familial case of RTS in two siblings of non-consanguineous parents. In our study of the case, we were able to carry out germline analysis, tracking of the segregation of the mutations, and genomic analysis of both tumors (somatic analysis).

Both siblings had cutaneous conditions, growth retardation, bone alterations, and osteosarcoma. Sporadic osteosarcoma typically occurs and is diagnosed at around the time of puberty and young adulthood, but osteosarcoma develops earlier in subjects with RTS, as happened with the two siblings described here^14,15^.

Our patients were treated with neo-adjuvant chemotherapy consisting of systemic Adriamycin and intra-arterial cisplatin followed by resection of the tumors with limb preservation. During MAP therapy, both patients suffered severe adverse events as a result of the toxicity of cytostatic drugs. This type of complication has already been described for RTS patients^16^ and can be regarded as an additional difficulty in providing treatment because reactions to toxicity can make it necessary to change treatment and a potential delay in chemotherapy and surgery. Despite growth retardation and the early onset of osteosarcoma, both patients were treated with limb-sparing procedures.

Osteosarcoma only rarely presents as part of a syndrome that includes a familial predisposition to cancer, and consequently, facial poikiloderma and the early age at which the tumor appeared were crucial pointers toward the right diagnosis. Genetic studies are not only mandatory for diagnostic confirmation of type II RTS but also enable appropriate surveillance of potential clinical complications. Thus these patients should be referred to a multidisciplinary pediatric team for the follow-up of traumatological, endocrinologic, dermatologic, dental, and ophthalmologic manifestations of the condition. In addition, once *RECQL4* mutations are identified in a proband, targeted genetic analysis and genetic counseling can be offered to first-degree relatives.

In the cases reported here, somatic analysis did not reveal any significant alteration, and therefore there was no requirement or criterion by which to personalize the treatment approach. In general, however, the deep sequencing of all types of pediatric tumors, even those coming from syndromes or from translocated tumors, which were traditionally considered as “monogenic” tumors, widens the possibilities of treatment and sheds light on diverse aspects of these diseases. Molecular alterations revealed by molecular diagnosis and somatic analysis of tumors can open up new avenues for research and advancement, be of use in clinical applications, serve as biomarkers, or constitute criteria for recruitment in clinical trials^17^.

Since osteosarcoma is rarely a clinical symptom of a syndrome, the recognition of the phenotypes associated with RTS is crucial for early diagnosis and early intervention. In fact, prompt recognition of RTS characteristics make it possible to anticipate future osteosarcoma, especially if a sibling has already been diagnosed with the disease. RTS patients should be referred to a multidisciplinary pediatric team for the follow-up of traumatological, endocrinologic, dermatologic, dental, and ophthalmologic manifestations of the condition.

## Methods

### Germline genetic analysis

DNA was obtained in a Maxwell® 16 System using the Maxwell® RSC Blood DNA Kit (Promega). DNA was quantified in a biophotometer and all exons and flanking intronic regions (+/−30 bp) of the *RECQL4* gene were PCR amplified and sequenced in a 3500 DX Genetic Analyzer from Applied Biosystems®. The obtained reads were compared to the consensus sequence of the gene (GenBank Accession Number: NM_004260.3).

### Somatic genomic analysis

DNA and RNA were extracted from non-decalcified tissue from the diagnostic true-cut biopsy samples of both siblings. Genomic analysis used the Oncomine™ Childhood Cancer Research Assay from Thermo Fisher; this is an NGS assay based on a 203-gene library. The assay consists on a large translocation/fusion RNA panel for 97 genes (with >1700 fusion isoform variants) and a DNA panel for 82 targets with comprehensive coverage of relevant mutations, 44 targets with full exon coverage (specifically tumor-suppressor genes), and 24 copy number variant targets (Supplementary Table 1). Note that the somatic NGS panel does not include the coding sequence of *RECQL4*, presumably because this gene is currently only useful for diagnosis of RTS syndrome.

### Ethics approval

The genomic analysis of our patients was part of their clinical management in the Clínica Universidad de Navarra. The detailed protocol and publication plan were explained to the parents who signed written informed consent to take part in the study.

### Reporting summary

Further information on research design is available in the Nature Research Reporting Summary linked to this article.

## Supplementary information

Supplementary Table 1

Reporting Summary

## Data Availability

The datasets generated during and/or analyzed during the current study, corresponding to the DNA genomic analysis, have been uploaded to the SRA database under BioProject accession PRJNA663851 (submission SUB8148545).
