# Supplementary material for: Somatic and germline analysis of a familial Rothmund–Thomson syndrome in two siblings with osteosarcoma
Source: NPJ Genom Med. 2020 Dec 4;5:51. doi: 10.1038/s41525-020-00160-x (PMC7718910; doi:10.1038/s41525-020-00160-x)
Supplement: Supplementary file 1 — Supplementary Table 1 [file 41525_2020_160_MOESM1_ESM.pdf]

Supplementary Table 1. Detailed description of the genes sequenced with the Oncomine™ Childhood Cancer Research Assay (Thermo Fisher). Gene coverage and read depth for all genes in both cases is shown. Highlighted in gray are genes sequenced at full CDS (coding sequence).

		% Coverage		Mean read depth (x)	
Gene	Bases in CDS	Case #1	Case #2	Case #1	Case #2
APC	8547	99.15	99.15	2388	1570
ARID1A	6878	99.8	99.8	2307	1822
ARID1B	6770	96.32	96.32	2563	1853
ATRX	7514	98.2	98.2	1221	556
CDKN2A	474	93.04	93.04	1440,	1526
CDKN2B	419	93.56	93.56	1547	1036
CEBPA	1078	100	73.84	673	523
CHD7	9031	100	100	2795	2173,96
CRLF1	1278	90.92	90.85	1798	1232,3
DDX3X	2003	99.2	99.2	1292	710,4
DICER1	5795	99.26	99.26	2705	1330
EBF1	1792	100	100	2068	1135
EED	1338	100	100	2385	883
FAS	1017	100	100	2409	942
GATA1	1247	100	100	1109	853
GATA3	1340	100	100	2229	1620
GNA13	1138	96.4	96.4	2537	1543
ID3	362	100	100	2850	1402
IKZF1	1567	100	100	2526	1675
KDM6A	4235	99.57	99.57	1191	785
KMT2D	16668	99.06	99.06	2489	1812
MYOD1	966	99.28	99.28	2085	1248
NF1	8514	98.99	98.99	2257	1365
NF2	1804	100	100	2521	1530
NSD2	4119	99.49	99.49	2482	1360
PHF6	1107	100	100	1180	537
PRPS1	964	100	100	1660	1042
PSMB5	485	100	100	3122	2077
PTCH1	4367	99.98	100	2531	2038
PTEN	1221	94.27	94.27	2196	860
RB1	2814	97.55	97.55	2148	845
RUNX1	1451	100	100	2228	1351
SMARCA4	5075	99.98	99.98	2262	1308
SMARCB1	1167	100	100	2297	1184
SOCS2	599	100	100	2437	1286
SUFU	1467	100	100	2662	1275

SUZ12	2236	92.13	92.13	2191	926
TCF3	1974	100	100	1945	1121
TET2	6018	100	100	2479	1644
TP53	1192	100	100	2140	1553
TSC1	3516	100	100	2391	1743
TSC2	5465	100	100	2006	1436
WT1	1579	94.93	94.93	2139	1503
XIAP	1500	96	96	1199	717
ABL1	3461	17.77	17.77	2363	2107
ABL2	3497	41.15	41.15	2818	1249
ACVR1	1539	64.52	64.52	2927	1078
AKT1	1456	7.49	7.49	1638	451
ALK	4892	17.13	17.13	2343	1799
ASXL1	4638	68.35	68.35	3085	2408
ASXL2	4320	73.94	73.94	3004	2134
BRAF	2319	30.62	30.62	2087	1618
CALR	1263	23.44	23.44	2381	4438
CBL	2737	20.68	20.68	2253	1775
CCND1	893	87.68	87.68	2151	1271
CCND3	640	26.41	26.41	3354	1578
CCR5	1060	11.51	11.51	1671	484
CDK4	919	46.68	46.68	1910	1111
CDK6	988	91.7	91.7	2450	1315
CIC	4847	12.65	12.65	1968	1167
CREBBP	7360	15.84	15.84	2187	1241
CRLF2	773	27.17	27.17	2318	1681
CSF1R	2940	7.79	7.79	1542	1119
CSF3R	2526	21.14	21.14	2429	1158
CTNNB1	2360	22.97	22.97	2105	1214
DAXX	2230	4.98	4.98	1831	1409
DNMT3A	2761	47.88	47.88	2391	1811
EGFR	3661	35.65	35.65	2289	1953
EP300	7276	4.23	4.23	1977	1379
ERBB2	3795	21.11	21.11	2035	1925
ERBB3	4057	22.21	22.21	2375	1470
ERBB4	3955	1.59	1.59	2894	1890
ESR1	1796	6.74	6.74	2132	1109
EZH2	2275	13.45	13.45	2321	1375
FASLG	850	62.82	62.82	2663	1146
FBXW7	2135	9.13	9.13	2375	1135
FGFR1	2486	40.79	40.79	2241	1396
FGFR2	2483	17.36	17.36	2048	1156
FGFR3	2438	19.98	19.98	1558	1757
FGFR4	2426	34.21	34.21	1704	930
FLT3	3006	10.15	10.15	2705	1346
GATA2	1448	18.92	18.92	2116	1300
GLI1	3332	32.89	32.89	1952	1462

GLI2	4774	24.86	24.86	1802	1195
GNA11	1087	14.54	14.54	1763	1042
GNAQ	1087	19.69	19.69	2435	1259
H3F3A	414	26.09	26.09	1458	779
HDAC9	3059	2.58	2.58	1777	951
HIST1H3B	412	32.04	32.04	2063	455
HRAS	574	36.24	36.24	1417	1081
IDH1	1253	8.3	8.3	2550	1469
IDH2	1370	9.56	9.56	2392	927
IGF1R	4125	24.24	24.24	2180	1122
IL7R	1388	15.27	15.27	2188	1554
JAK1	3489	46.6	46.6	2585	1520
JAK2	3422	55.32	55.32	2319	1035
JAK3	3398	57.09	57.09	2106	1404
KDM4C	3192	3.85	3.85	1513	673
KDR	4101	7.29	7.29	2058	2703
KIT	2952	18.5	18.5	2132	1168
KRAS	571	51.66	51.66	2046	1020
MAP2K1	1193	28.92	28.92	2261	1310
MAP2K2	1214	9.14	9.14	2474	1171
MDM2	1505	22.33	22.33	2019	1061
MDM4	1483	62.44	62.44	2222	868
MET	4247	25.81	25.81	2259	1252
MPL	1920	4.17	4.17	1256	3806
MSH6	4093	9.06	9.06	2359	979
MTOR	7707	7.12	7.12	1927	1379
MYC	1368	34.8	34.8	1993	2645
MYCN	1397	9.52	9.52	1711	1105
NCOR2	7592	6.47	6.47	1549	1008
NOTCH1	7702	41.17	41.17	2083	1390
NPM1	896	12.95	12.95	2502	672
NRAS	574	49.3	49.3	1767	2160
NT5C2	1703	16.15	16.15	1795	727
PAX5	1186	56.75	56.75	2366	1611
PDGFRA	3292	36.73	36.73	2463	1507
PDGFRB	3343	3.89	3.89	1407	615
PIK3CA	3227	33.25	33.25	2376	1381
PIK3R1	2190	44.25	44.25	2210	828
PPM1D	1824	21.98	21.98	3365	1384
PTPN11	1797	14.64	14.64	1874	944
RAF1	1963	15.44	15.44	2084	1117
RET	3365	10.79	10.79	2022	1173
RHOA	586	47.61	47.61	2964	1236
SETBP1	4796	2.42	2.42	2524	1393
SETD2	7716	8.98	8.98	2127	763
SH2B3	1735	26.05	26.05	1471	678
SH2D1A	391	89.26	89.26	1236	753

SMO	2376	17.63	17.63	1975	1689
STAT3	2336	19.31	19.31	2635	1647
STAT5B	2382	9.11	9.11	2201	889
TERT	3415	1.46	1.46	500	894
TPMT	746	23.73	23.73	1953	602
USP7	3340	18.2	18.2	2263	1009
ZMYM3	4137	11.58	11.58	982	559
